# Editorial: From static storage to *ex vivo* reconditioning of donor grafts

**DOI:** 10.3389/frtra.2023.1272440

**Published:** 2023-09-01

**Authors:** Kojiro Nakamura, Gabriel C. Oniscu, Hirofumi Hirao

**Affiliations:** ^1^ Department of Surgery, Kyoto University, Kyoto, Japan; ^2^ Department of Clinical Science, Intervention and Technology, Karolinska Institute, Stockholm, Sweden

**Keywords:** transplantation, preservation, *ex vivo* machine perfusion, subzero organ preservation, mitochondria

**Editorial on the Research Topic** From static storage to *ex vivo* reconditioning of donor grafts

We are delighted to accept six articles on the Research Topic of “*From Static Storage to Ex Vivo Reconditioning of Donor Grafts*” which have received widespread interest. Organ transplantation remains the only treatment option for patients with end-stage organ failure. The discrepancy between the need and the number of organs available results in a transplant waiting list of over 100,000 in the United States alone, which is generally considered the tip of the iceberg for those in need. Although there has been rapid growth in organ transplantation, the need for more transplantable grafts remains desperately high (Ozgur et al.).

Thoracic organ transplantation are highly regarded as gold standard treatments for patients suffering from heart failure or chronic end stage lung conditions. Improved preservation techniques beyond static storage have shown great potential to lengthen the current period of viability of thoracic organs while outside the body, promising better utilization rates, increased donation distance, and improved matching of donors to recipients. *Ex vivo* organ perfusion can also facilitate some novel therapeutic strategies viable, and the combination with reconditioning therapies endeavors to better improve functional preservation of organs in addition to making more organs suitable for transplantation. Wagner et al. summarized important concepts and research regarding thoracic organ machine perfusion in combination with reconditioning therapies. In the future, pathophysiology mediated during the transplant process will be able to be completely reversed by therapeutic options, permitting organs to be not only reconditioned from unsuitable to transplantable, but also acting as a standalone treatment modality (Wagner et al.).

Pancreas transplantation is the only curative treatment for type-1 diabetes that maintains normoglycemia thus avoiding complications arising from poor glycemic control. At present, the *ex vivo* normothermic machine perfusion technique allows organ monitoring and estimation of its quality before transplantation, unlike clinical pancreas transplantation, for which there are few experimental and pre-clinical studies published. Outcome measures in current studies of *ex vivo* normothermic machine perfusion -treated pancreases include blood flow, blood gas analysis, mean arterial pressure, endocrine function under glucose stimulation, amylase and lipase levels, production of pancreatic secretion, metabolic markers, and histologic analysis. With the limited publications in pancreas *ex vivo* normothermic machine perfusion, there are no clear, approved, and accurate conditions to enable its implementation on a routine basis in pancreatic clinical settings. Significant refinements to the technique and rigorous analysis of data from larger studies are urgently required (Ferrer-Fàbrega et al.).

Mitochondrial activity and their capacity for ATP synthesis make them an important mediator of cellular injury, immune activation, and cell death during each phase of organ transplantation. Taylor et al. discuss the interplay of donor brain death (BD), mitochondrial dynamics, and impact on allograft function. Targeting mitochondrial responses at each point of the axial dynamics is an opportunity to blunt the donor response to BD and its impact on potentially transplantable organs (Taylor et al.). Quiring et al. focused on the cold-induced mitochondrial fission in endothelial cells and assessed in a cell culture model at which rewarming temperature mitochondrial re-fusion, ATP production and cellular regenerative capacity return after cold incubation. Their data suggested that for optimal recovery of mitochondrial network and function during reconditioning after cold storage, rewarming temperatures of ≥25°C (best of 37°C) are preferable from a mechanistic point of view (Quiring et al.). Hypothermic machine perfusion is the standard of care for kidneys originating from donation after circulatory death, whereas the evidence of hypothermic oxygenated machine perfusion (HOPE) is still evolving. By using a porcine kidney perfusion model, Silva et al. measured flavin mononucleotide (FMN), mitochondria derived fragments, in the perfusate, and then found the correlation between FMN quantification and pre-existing kidney graft injury. Real-time FMN measurement during HOPE may be an objective assessment tool to accept high-risk kidneys for transplantation while minimizing post-transplant dysfunction (Silva et al.).

In addition to the machine perfusion, next generation technologies have the potential to further extend the limits and open the door to banking organs, including supercooling, partial freezing, and nanowarming. The likely near-term trajectory is that these techniques will be developed towards clinical testing in order of technological readiness levels. It is possible to envision a future where there are multiple preservation techniques employed based on need: for ideal organs with a recipient in close proximity, static cold storage can be perfectly adequate; for organs that need repair, some form of perfusion would allow *ex vivo* treatment, which would further allow allocation of organs in an expanded area. For international exchanges and allocation and short-term banking in the order of months, high subzero organ may well would be the solution. For very long-term banking, which would allow off-the-shelf readiness for organs, low subzero approaches would be the real solution. Isochoric approaches could either boost one of these or be completely enabling by, for instance, allowing a specific approach to be practical without the need for using toxic levels of controlled rewarming. Whether these options can simultaneously be viable commercially will depend on cost, practicality, and efficacy, which are yet to be determined (Ozgur et al.).

Static cold storage has been the backbone of organ preservation, allowing transplantation to establish itself as a treatment option worldwide for patients with end-stage organ dysfunction. Meanwhile, the ice-box strategy is associated with various limitations including inevitability of ischemia-reperfusion injury, difficulty in assessing graft function, limited opportunity for organ repair. *Ex vivo* organ perfusion has recently emerged as a promising strategy not only to alleviate graft injury but also to serve as an extracorporeal platform for organ reconditioning and graft assessment. Under this Research Topic, we have gained updated understanding of *ex vivo* organ perfusion/preservation as well as the next generation technologies. Future studies are warranted to establish tailored *ex vivo* organ perfusion strategies and develop the subzero techniques.

